# Alzheimer’s disease neuropathological change and loss of matrix/neuropil in patients with idiopathic Normal Pressure Hydrocephalus, a model of Alzheimer’s disease

**DOI:** 10.1186/s40478-019-0748-9

**Published:** 2019-05-29

**Authors:** Sylwia Libard, Irina Alafuzoff

**Affiliations:** 1 Department of Immunology, Genetics and Pathology, Uppsala University, Rudbeck Laboratory, Dag Hammarskjölds väg 20, 751 85, Uppsala, Sweden; 2 Department of Pathology, Uppsala University Hospital, Uppsala, Sweden

**Keywords:** Alzheimer’s disease Neuropathologic change, Idiopathic Normal pressure hydrocephalus, Neuronal loss, Matrix/neuropil, Immunohistochemistry

## Abstract

Here, we assessed unique brain tissue samples, obtained from living subjects with idiopathic Normal Pressure Hydrocephalus (iNPH). Our cohort of 95 subjects with age ranging from 75 to 79 years, displayed a high prevalence of β-amyloid (Aβ) and hyperphosphorylated τ (HPτ) pathology (63 and 61%, respectively) in a frontal cortex biopsy obtained during shunt operation. These lesions, i.e., Alzheimer’s Disease Neuropathologic Change (ADNC), increased within 5 years and were more frequent in females. The extent of HPτ pathology was sparse, primarily seen as neurites and stained dots. Noteworthy, concomitant pathology was seen in 49% of the whole cohort, indicating a severity of ADNC corresponding to a low/intermediate level following the current recommendations. This observation is predictable as based on previous publications a substantial number of subjects with iNPH over time develop AD. Thus, iNPH can be considered as a model of AD. We noted a surprisingly remarkable neuronal preservation assessing Neuronal Nuclei (NeuN) in parallel with a substantial depletion of matrix/neuropil. This finding is intriguing as it suggests that loss of matrix/neuropil might be one of the first lesion of ADNC but also a hallmark lesion of iNPH. The latter observation is in line with the enlarged ventricles, a cardinal feature of iNPH. Furthermore, a positive correlation was observed between the extent of Aβ and NeuN but only in females indicating a neuronal preservation even when Aβ pathology is present. The assessment of a surgical biopsy as described here is certainly informative and thus it is surprising that a neuropathologic assessment in the setting of iNPH, while inserting a shunt, is seldom performed. Here, we observed ADNC and surprisingly remarkable neuronal preservation in a substantial number of iNPH subjects. Thus, these subjects allow us to observe the natural course of the disease and give us an opportunity for intervention at the earliest stages of AD, prior to severe neuronal damage.

## Introduction

The idiopathic normal pressure hydrocephalus (iNPH) is a neurological condition affecting elderly patients, with symptoms including gait disturbance, cognitive impairment (CI), and urinary incontinence [42]. The diagnosis is based on the existence of the above given symptom triad, detection of enlarged ventricles on imaging, and pathological cerebrospinal fluid (CSF) dynamic tests [42]. Up to date, no characteristic neuropathologic lesion or post mortem (PM) applicable neuropathological consensus criteria are available for iNPH.

Previous publications have shown that a substantial number of subjects with iNPH display, in a minimal cortical biopsy obtained during ventriculoperitoneal shunt (VPS) insertion, hallmark lesions of Alzheimer’s Disease (AD). The AD neuropathologic changes (ADNC) include β-amyloid (Aβ) and hyperphosphorylated τ (HPτ) aggregates, primarily seen in the gray mater [3, 5–7, 13, 23, 25, 31, 35, 40]. Noteworthy, a substantial number of subjects with iNPH develop clinical dementia over time, particularly dementia of the AD type [20]. In line with the above, concomitant ADNC have been seen in PM neuropathological examination in a subset of patients with clinical diagnosis of iNPH [11, 22, 25].

The treatment of choice of iNPH is insertion of a VPS that can, in some cases, reverse the symptoms by normalizing the CSF flow in the cerebral cavities [42]. While shunting, a catheter is inserted through the brain parenchyma, usually the right frontal lobe, into the lateral ventricle and the opposite tip of the catheter is directed towards the peritoneal space. There is a shunt valve between the catheter ends, opening when the CSF pressure is excessively high, aiming to reach a physiologic equilibrium and normalize the CSF flow within the brain [42]. Thus the VPS is inserted to continuously alleviate eventual increase in the CSF pressure. Interestingly, the presence of ADNC in the biopsy has been associated with a lack of VPS response and with worse prognosis after the VPS surgery [1, 5, 20, 31].

There are a few centers in the world that have obtained a cortical biopsy during the VPS insertion; similarly, there are a few reports on PM neuropathologic finding in subjects with clinical diagnosis of iNPH [5, 11, 13, 22, 23, 25, 31, 35].

AD is the most common neurodegenerative disease causing dementia [4]. The cardinal symptoms are CI and/or memory loss, considered to be caused by a progressive synaptic and neuronal damage [28, 33, 37, 38]. Several reports indicate that the synaptic loss and neuronal damage are associated with progressive accumulation of Aβ and HPτ in the brain tissue [18, 26, 33, 37, 38]. Noteworthy, some studies describe cell loss in the early stages of AD [16], whereas others state that neurons can survive and function even displaying HPτ, also at a late stage of the disease [10]. However, it should be noted that stepwise progression of ADNC is observed in a substantial number of unimpaired subjects prior to the clinical AD syndrome but also in individuals that never progress to dementia [8, 9, 14]. Thus, the assessment of synaptic loss and neuronal loss in association with ADNC is more complex than anticipated.

Most of the studies addressing synaptic loss and neuronal loss in AD are performed on PM brain tissue, animal models, or cell lines. Regarding iNPH, there are no animal models or cell lines; moreover, the human brain tissue to be assessed is scanty. Thus, to our knowledge, these alterations have not been addressed.

The purpose of this study was to assess ADNC and neuronal loss, applying sensitive immunohistochemical (IHC) technique in surgical biopsies from frontal cortex of patients with clinical diagnosis of iNPH. The benefit of this approach is that we are investigating human tissue, i.e., a surgical sample, which is well preserved, lacking all changes that are related to PM events, including agonal state, PM delay, and long fixation time. The pitfall is the minimal size of the biopsy, but this issue was considered negligible as the objective was to assess defined alterations within a defined area.

## Material and methods

### Ethical statement

The study was approved by the regional Ethical Committee of Uppsala, Sweden #2013/176, updated 2016. All the subjects included have given their informed consent for the use of their tissue samples for clinical and scientific purposes.

### Study subjects

The study was carried out on brain biopsies obtained for diagnostics, from patients diagnosed with iNPH and operated on at the Neurosurgery Department at the Uppsala University Hospital (UUH), Sweden, during 2010–2016. In total, brain samples from 364 subjects were identified at the Pathology Department, UUH. In addition, the subject had to be within the age range of 75 to 79 years, as the age is known to significantly influence all the alterations to be assessed [14, 32]. In total, 107 subjects were identified. Seven of these subjects displayed only white matter in their biopsies and were thus excluded. An additional five individuals were excluded due to the loss of gray matter during the processing of the tissue. Thus, brain tissue samples from 95 subjects remained for the final analysis. In addition, a set of samples based on the outcome in one of the neuronal markers were selected to be IHC stained with antibody to synaptophysin (SYP).

### Brain samples

The diagnostic brain biopsies were obtained during the VPS operation, as previously described [13, 23, 42]. The samples were taken from the right frontal lobe, more exactly, within an area of the superior- and medial- right frontal gyri. The biopsies were then fixed in 10% neutral buffered formalin (4% formaldehyde) for 24 h at room temperature and processed into paraffin (Histowax from Histolab) blocks. The blocks were sectioned on the microtome from Thermo Scientific (Microm HM 355S) into 4 μm thick sections, which were placed on Super Frost slides for standard Hematoxylin-Eosin and Super Frost Plus slides for IHC stainings.

### Immunohistochemistry

The IHC stainings were carried out automatically or manually. The automated stainings were performed on the Dako Autostainer Plus (DakoCytomation, Glostrup, Denmark) using the Dako EnVision Flex detection system (DakoCytomation, Glostrup, Denmark), according to the manufacturer’s instructions. The Bright Vision detection system (IL Immunologic, Duiven, The Netherlands), with a Romulin AEC for antigen detection (BioCare Medical, Pacheco, CA, U.S.A.), was used for the manual stainings. The antibodies (Ab) used and the pretreatment strategies applied are listed in Table 1.

**Table 1:** Immunohistochemical stains

Antibody	Clone	Company/Code	Dilution	Pretreatment
Amyloid β (Aβ)	6F/3D	Dako-Agilent/M0872	1:50	98–100% FA
Embryonic lethal abnormal visual system proteins (nELAV) 3 and 4 human homolog HuC/HuD (HuC/HuD)	16A11	ThermoFisher Scientific/A-21271	1:2000	ac, CB
NEUronal Nuclei (NeuN)	A60	Milipore/MAB377	1:2000	CB
Hyperphosphorylated (Ser202/Thr205) τ (TAU8)	PHF-TAU-AT8	Fisher Sientific-Invitrogen/MN1020	1:1000	
Synaptophysin (SYP)	SY38	Dako-Cytomation/M0776	1:50	CB

Dako Autostainer Plus (Dako Cytomation) was used for Aβ, Tau8 and SYP, while the HuC/HuD and NeuN were carried out manually, incubation at room temperature for 1 h. Autoclave (ac), formic acid (FA), citrate buffer pH 6.0 (CB)

### The analysis of the samples

Primarily, all biopsies were assessed using light microscopy (Olympus BX45) at magnification × 20 to × 400. The samples were then scanned into digital slides, saved as ScanScope Virtual Slide (svs) format, with Aperio AT2 (Leica Biosystems) scanning system in 20x magnification. For the morphometric image analyses, the Aperio ImageScope software (Leica Biosystems) was used, applying the positive pixel count algorithm (version 9.1) for quantification of the immunoreactivity (IR). All parameters were preset in the software, except “The Intensity Threshold (Upper Limit) of WEAK positive pixels,” which was increased from 220 to 255.

A color code was used to visualize the staining intensity, where negative pixels were blue, the week staining was visualized in yellow, moderate in orange, and the strong staining intensity was brown. The algorithm was applied on the gray matter area in each biopsy. The molecular layer of the gray matter and vessels with cerebral amyloid angiopathy (CAA) were not included in the analysis when present in the sample. The IR positive pixels were calculated in the assessed gray matter area and transformed to stained area in mm^2^. The number of IR pixels, i.e., pixels of given intensity, counted in the analysis was dependent of the staining quality and the compartment of the structure that expressed the protein of interest. The sum of all the positive pixels, i.e., weak, moderate, and strong, was assessed for Aβ. In HPτ - and embryonic lethal abnormal visual system proteins 3 and 4 human homolog HuC/HuD (HuC/HuD) stainings, the moderate- and strong positive pixels were included. Only the strong positive pixels were counted for the Neuronal Nuclei (NeuN) and SYP staining. The final quantification of the IR in the tissue is given as a stained area fraction (SAF), a ratio between the stained IR area per total area analyzed in the biopsy × 100.

### Statistical analysis

IBM SPSS statistics (version 25) was used for the statistical analyses. Here, we used non-parametric tests such as Mann Whitney U test (MWU) and Kruskal Wallis test (KWT) to assess the differences between the genders and different age groups. Spearman’s rho two tail test was used to define the correlation between the studied variables.

## Results

Ninety-five individuals, 45 females and 50 males, between the ages of 75 to 79 were included in this study. The mean age ± standard error of means (m ± SE), at biopsy for the whole cohort was 77.03 ± 0.14 years, for females 76.96 ± 0.21, and for males 77.10 ± 0.2 years. No significant age difference was observed between the genders (MWU). The total area analyzed in the frontal cortex biopsies, represented by the gray matter, varied from 0.16 to 38.00 mm^2^ (7.69 ± 0.33 mm^2^).

Microscopic evaluation displayed no inflammatory processes, tumors, or extensive bleeding, alterations that might affect the interpretation of the pathology to be assessed within the tissue. CAA was noted in four out of the 95 cases (4%), not included in the SAF values below. The presence of ADNP, i.e., Aβ and HPτ, was verified by light microscopy in × 20 – × 400 magnification. Light microscopic assessment of the staining outcome while applying the neuronal markers NeuN and HuC/HuD confirmed the neuronal compartmentalization and SYP localization into neuropil.

**Fig. 1:**
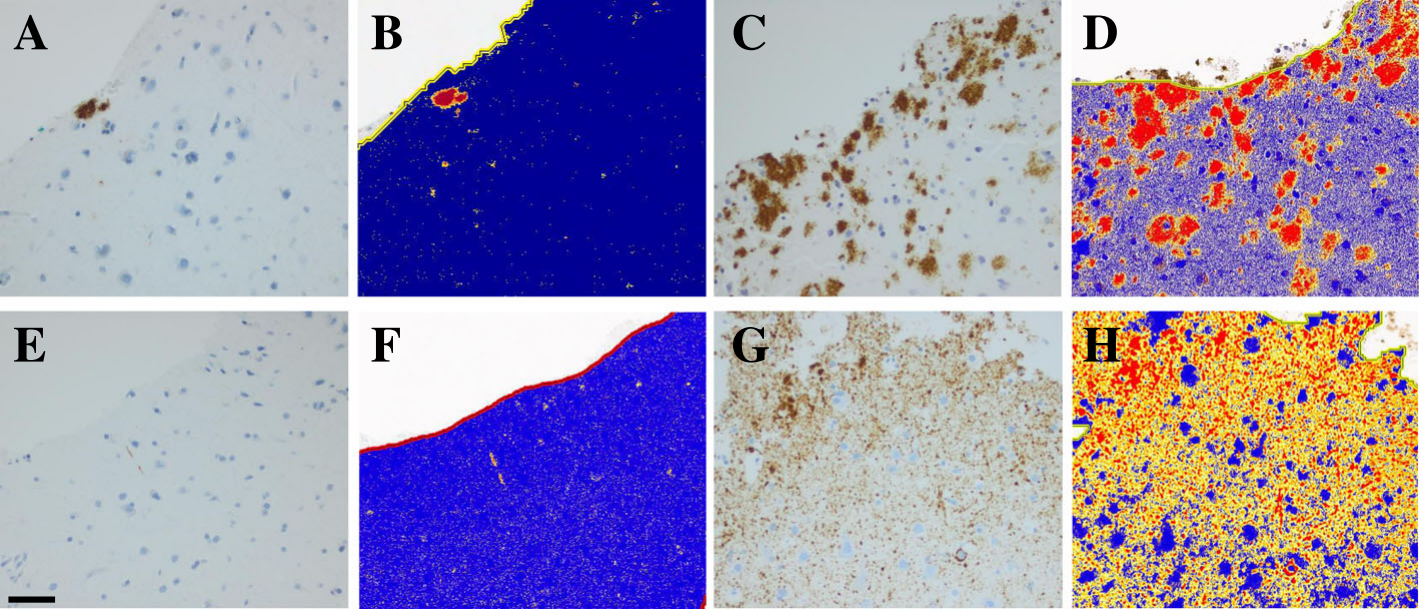
Photos of immunohistochemically (IHC) stained sections of brain biopsies from the frontal cortex. In **a**-**d**, the IHC outcome when applying antibodies directed to the β-amyloid (Aβ) and in E-H applying hyperphosphorylated τ protein (HPτ). In **b**, **d**, **f**, and **h**, the results of positive pixel count analysis, where negative pixels are blue, weak stained positive pixels are yellow, moderately stained pixels are orange, and strong positive pixels are brown. In **a**-**b**, Aβ extent in a case with stained area fraction (SAF) of 1%; in C-D Aβ extent SAF 27%; E-F HPτ extent SAF 0.05% and in G-H HPτ extent SAF 19%. Bar 50 μm

IHC/Aβ was seen in 60 out of the 95 (63%) biopsies: 32 of 45 (71%) females and 28 of 50 (56%) males. The extent of the IHC/Aβ varied from a single aggregate to multiple diffuse and compact aggregates within the tissue, with the SAF ranging from ~ 1 to 27% (Fig. 1a-d). The SAF/Aβ was significantly influenced by age, but the significant association was not observed in females (Table 2). IHC/HPτ was found in 58 (61%) biopsies: 31of 45 (69%) females and 27 of 50 (54%) males. HPτ pathology increased from a single granule or thread to multiple tangles in the neurons and/or abundant neurites within the neuropil, with the SAF ranging from 0.02 to 19% (Fig. 1, e-h). The SAF of HPτ was less than 1% in as many as 47 of the 58 (81%) samples. The SAF/HPτ was significantly influenced by age but only in the whole cohort (Table 2). Concomitant IHC/Aβ- and IHC/HPτ- pathology was observed in 47 out of 95 (49%) subjects in our cohort; moreover, the SAF of these pathologies showed a significant correlation, for both men and women (Table 2).

**Table 2:** Spearman’s rho correlations and significance ^p^. Correlation is significant at the 0.01 level (2-tailed) is given in bold

	All	Female	Male
Number	96	45	50
Age/Aβ	0.19^0.07^		0.26^0.07^
Age/HPτ	0.18^0.08^		
Age/NeuN	**0.49** ^**0.000**^	**0.42** ^**0.004**^	**0.51** ^**0.000**^
Aβ/ HPτ	**0.58** ^**0.000**^	**0.64** ^**0.000**^	**0.47** ^**0.001**^
Aβ/NeuN		0.28^0.06^	
NeuN/HuC/HuD	**0.27** ^**0.010**^		0.26^0.06^

*Aβ* Amyloid β, *HPτ* Hyperphosphorylatedτ, *NeuN* NEUronal Nuclei, *HuC/HuD* Embryonic lethal abnormal visual system proteins (nELAV) 3 and 4 human homolog HuC/HuD

**Fig. 2:**
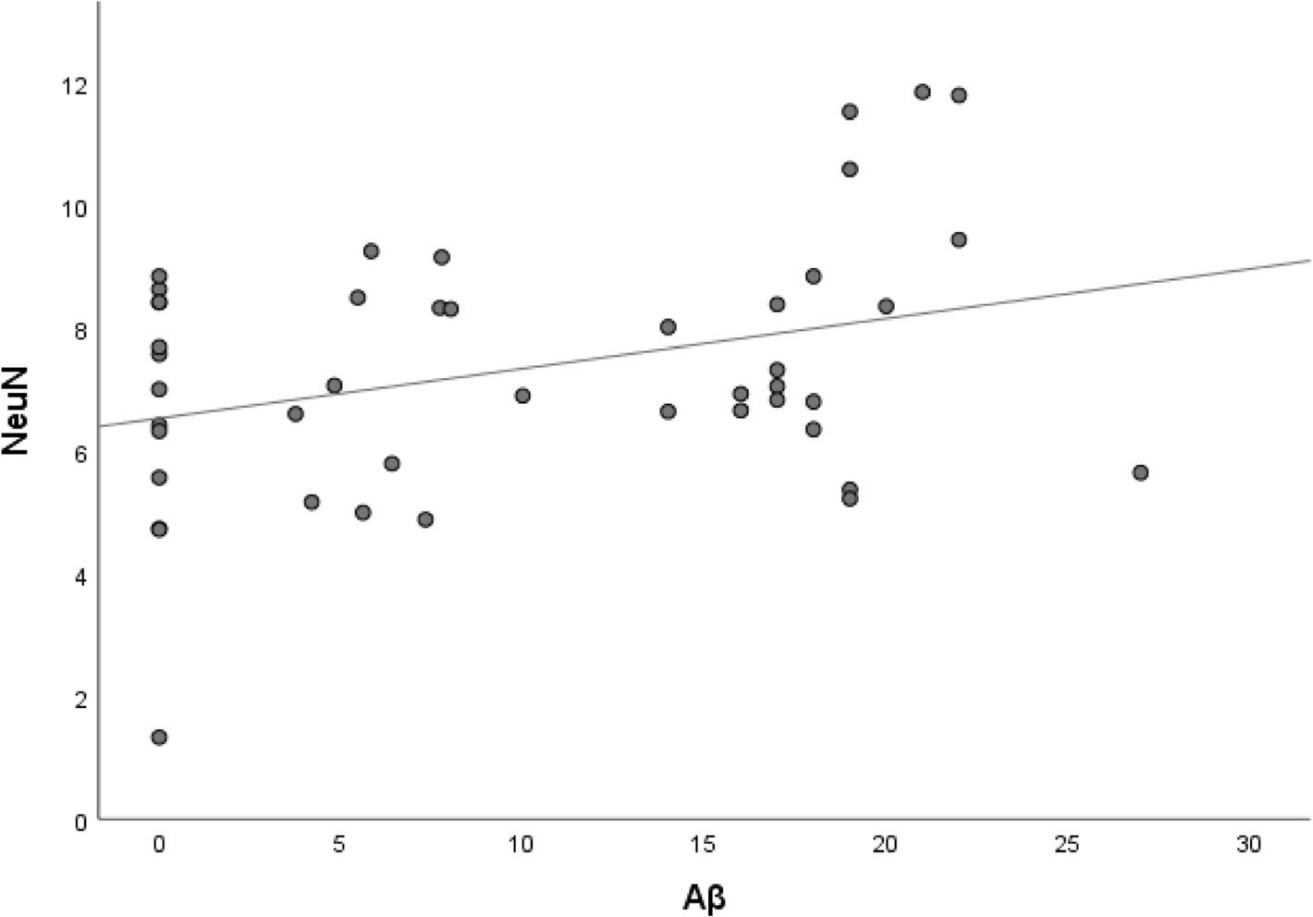
Dot plot to illustrate the correlation between stained area fraction (SAF) of Neuronal Nuclei (NeuN) and SAF of β-amyloid (Aβ) in 45 females with idiopathic Normal Pressure Hydrocephalus. Spearman’s rho correlation coefficient = 0.28, *p* = 0.06

A significant increase of NeuN was observed with increasing age. A significant correlation was observed between the two neuronal markers NeuN and HuC/HuD (Table 2). A positive correlation (*p* < 0.06) was observed between Aβ and NeuN, but only in females (Fig. 2).

When comparing the age groups, the SAF/NeuN was significantly higher in the oldest when compared with the youngest subjects (KWT *p* = 0.000). Contrary to the above, the age did not influence the SAF of HuC/HuD (MWU/KWT).

**Fig. 3:**
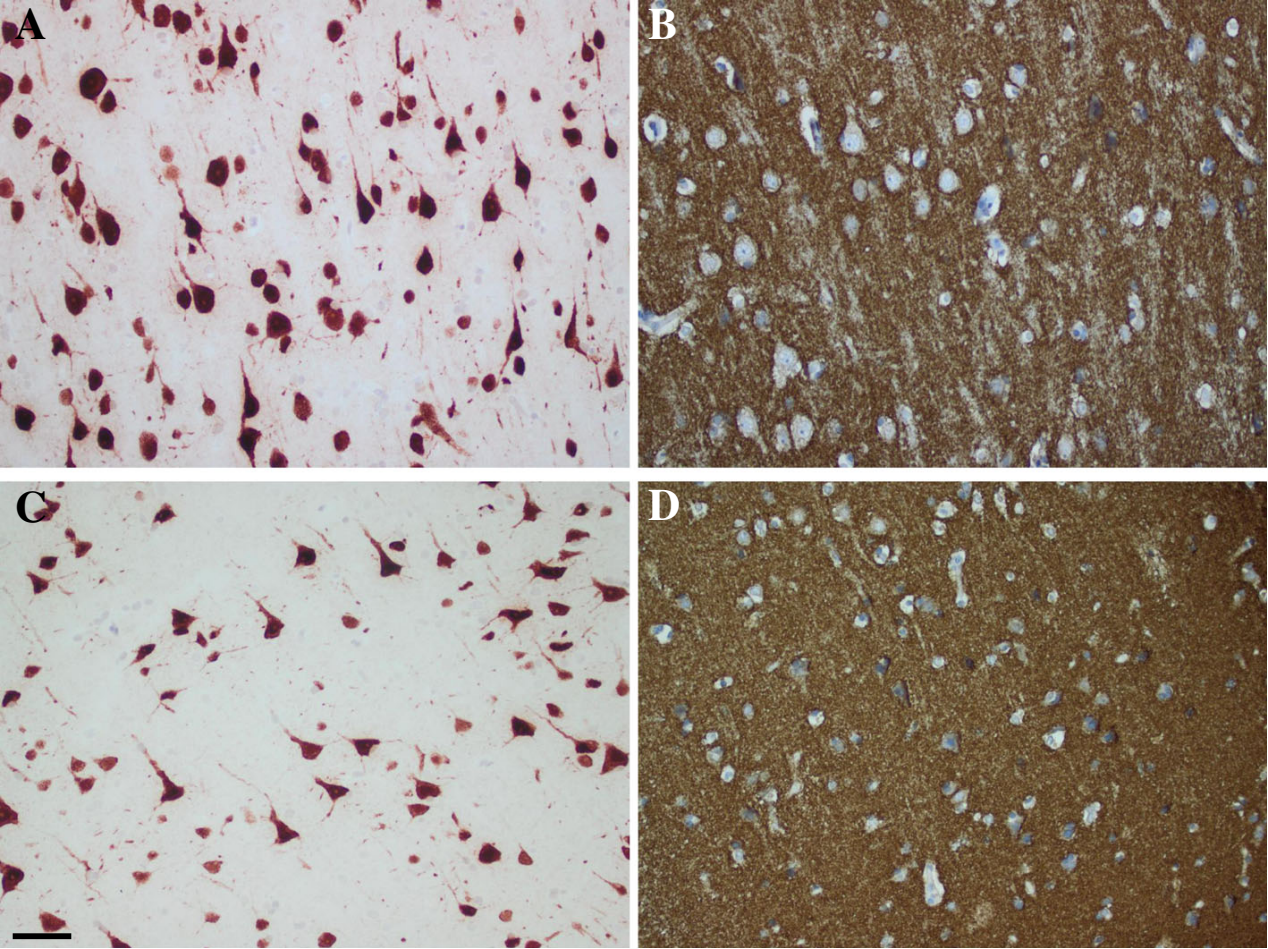
Photos of immunohistochemically (IHC) stained sections of brain biopsy from the frontal cortex, applying antibody directed to the Neuronal Nuclei (NeuN) or synaptophysin (SYP). In **a** and **c**, NeuN stained area fraction (SAF), in A 10% and C 5%. Note the significant difference in neurons to be seen in the same area. In **b** and **c**, SYP SAF in B 86% and in D 90%. Note in **b**, the loss of granular staining of SYP when comparing with **d**. Bar 50 μm

The m ± SE of SAF of the selected four cases for the synaptic marker SYP was 95.31 ± 0.36%. The two samples with the lower values of the SAF/NeuN (~ 5%) displayed a SAF/SYP value (90 and 91%) that was slightly higher than the SAF/SYP value (86 and 89%) observed in the two samples with a relatively high value of SAF/NeuN (~ 10%), as shown in Fig. 3.

The SAF of assessed proteins, in the whole cohort and in the genders, is presented in Table 3. There was a significant difference in the SAF/HPτ between the genders, where females displayed higher values of SAF/HPτ. The females also had higher SAF/Aβ values (borderline significant). Furthermore, a borderline difference was observed for SAF/Huc/HuD, whereas no difference was seen between the genders for the SAF/NeuN.

**Table 3:** Stained area fraction (SAF) in percent, for the antibodies used, when comparing females and males. Significance level is 0.05 is given in bold

	Value	All	Females	Males	Statistics/MWU
Number		95	45	50	
Aβ	m ± SE	8.30 ± 0.84	9.93 ± 1.25	6.83 ± 1.11	*p* = 0.060
HPτ	m ± SE	1.04 ± 0.32	1.51 ± 0.57	0.62 ± 0.33	***p*** **= 0.022**
NeuN	m ± SE	7.50 ± 0.23	7.34 ± 0.30	7.68 ± 0.35	ns
HuC/HuD	m ± SE	8.74 ± 0.29	8.38 ± 0.42	9.08 ± 0.40	*p* = 0.060

*Aβ* Amyloid β, *HPτ* Hyperphosphorylatedτ, *NeuN* NEUronal Nuclei, *HuC/HuD* Embryonic lethal abnormal visual system proteins (nELAV) 3 and 4 human homolog HuC/HuD, Syp, Synaptophysin, *MWU* Mann Whitney U test, the significance level is at 0.05; m ± SE, mean ± standard error of mean

## Discussion

A substantial number of cortical biopsies studied here, obtained from 95 subjects with iNPH, exhibited ADNC, with Aβ in 63 and HPτ in 61%. The frequency of subjects with Aβ is in line with previous reports whereas the percent of subjects with HPτ is significantly higher [5, 13, 23, 25, 31, 35]. One explanation to these discrepant results is the age of the cohort investigated. We chose to include subjects within the age range of 75–79 years, compared to other studies that included subjects within an age range from 28 up to 87 years [23]. We excluded the oldest to be confident that Age Related Tau AstroGliopathy (ARTAG) pathology was not present [21].

Another factor that might have influenced the finding of a high SAF/HPτ in our cohort is the methodology. Visualization of HPτ applying IHC and automatic platform is currently relatively secure, whereas the assessment of IR might vary. Here we used morphometric method and assessed all pathology disregarding the size of the alteration, i.e., also tiny granules were included. The SAF/HPτ in our study varied from 0.02 (only few grains) to a SAF value of 19%. A low value of SAF (0.05) is pictured in Fig. 1e-f, visualizing both the sparse extent and focal distribution of IR that might be disregarded without the use of imaging technique. To obtain a result in line with previous reports (10% of subjects with HPτ pathology), all subjects with sparse HPτ/SAF (0.02–1.5%) should have been excluded in our cohort [23]. In summary, three aspects that have influenced the outcome are the age of the cohort, method used to visualize the lesion and assessment strategy implemented.

Our observation of a relatively high prevalence of Aβ- and HPτ- pathology (63 and 61%) is in line with the biology of ADNC, known for increases with chronological age [9, 14]. Noteworthy, even in a relatively narrow age span of 5 years, from 75 to 79 years, the extent of both HPτ and Aβ correlated with the age at a borderline level.

In line with what has been reported in PM studies, a significant correlation was observed between the extent of hallmark lesions of AD, Aβ, and HPτ. The two protein alterations of ADNC progress, following distinctive neuroanatomical regions; Aβ, starting in the cerebral cortex, progresses through the limbic regions of the brain toward the cerebellum, whereas HPτ is seen initially in the subcortical areas, following in the limbic structures and finally in the neocortex [3, 6, 7, 40]. Thus, when both these alterations, i.e., ADNC, are observed in the biopsy obtained from the cerebral cortex, the finding suggests a stage of ADNC pathology corresponding to at least low/intermediate level, following the current staging strategy [17, 27]. These observations of a substantial number (~ 60%) of iNPH displaying low/intermediate level of ADNC is in line with a previous report indicating that 50% of subjects with iNPH develop dementia over time, presumably of the AD type [20].

Surprisingly, when assessing the neuronal marker NeuN, we noted an increase in the SAF with age, i.e., we observed a higher number of neurons within a defined area, i.e., more neurons in relation to the matrix/neuropil. This finding suggests that in our cohort of iNPH subjects, a significant alteration not previously reported is depletion of the matrix/neuropil. One explanation for this outcome could be consolidation of neuropil due to an eventual degenerative process, including loss of neuronal- and glial processes or depletion of extracellular matrix proteins. Both a loss of neuronal processes and a depletion of matrix proteins have been previously reported to be observed in subjects with AD but not in subjects with iNPH [36–38]. Thus, it is not clear whether this observation, i.e., depletion of the matrix/neuropil is due to the ADNC alterations, seen in a high number of our cohort, or whether it is an alteration related to iNPH. Interestingly the NeuN correlated with Aβ at a borderline level but only in females, and this finding might be due to a more severe depletion of neuropil, i.e., neuronal processes and matrix proteins in females parallel with the increase of Aβ (Fig. 2).

Synaptic density, a measure of the depletion of the neuropil, has been studied for many decades and is reported to occur in normal aging and is accelerated in different neurodegenerative diseases, particularly in AD [26, 37, 38]. Synaptic damage is seen early in the disease process; furthermore, synapses are supposed to be involved in the propagation of altered proteins, and the loss of synapses is suggested as being causative regarding CI [18, 33, 37, 38]. The changes in the synapses are suggested as being induced by ADNC, but the exact process is yet to be discovered [18, 33, 37, 38].

Here, we selected only a few samples to assess the SAF/SYP. The selection was based on neuronal counts, two with the highest and two with the lowest SAF/NeuN. These four cases were selected, disregarding the ADNC pathology or age. We noted a slight decrease in the SAF/SYP in patients with a higher neuronal density. This finding suggests that a depletion of the matrix might be an early change, preceding a neuronal loss, i.e., the processes are altered prior to the cell loss. This observation is certainly questionable due to the low number of subjects assessed, but it emphasizes that studies on matrix versus neuronal somata in degenerative diseases are certainly warranted.

Based on our and previous results, non-invasive-, in vivo-, techniques to assess ADNC in iNPH is certainly of interest. Currently, there is the Positron Emission Tomography (PET), technique applying specific tracers to assess the deposits of Aβ and HPτ in the brain [19, 29]. The most widely used is Carbon-11 labelled thioflavin-T derivate, Pittsburgh compound B [^11^C] PIB, to visualize Aβ, considered as being a reliable marker of Aβ pathology even in biopsied patients with iNPH [19, 34, 39]. In contrast, a HPτ selective tracer [^18^F]THK-5117 did not reflect the HPτ pathology in the cortical biopsies from iNPH subjects [24, 29]. The latter observation is due to the extremely low load of HPτ pathology as observed here.

The low SAF/HPτ and the observation of high neuronal density in a setting of iNPH suggest that neuroprotective-, antiapoptotic-, and HPτ targeting therapies may benefit these patients by delaying or slowing down the neurodegenerative process that eventually will lead to AD, as previously reported [2, 12, 20, 25, 41, 43]. Furthermore, there is an urgent need to learn more about the constitutions of the matrix to eventually identify new targets for therapy.

Two-thirds of all patients affected by AD are females; thus, the female gender, next to age and apolipoprotein E status, has been reported as being one of the risk factors of AD [4]. In line with the above, in our cohort of subjects with iNPH, a higher number of females displayed ADNC when compared with men. This was noted particularly for HPτ (both incidence and extent) but also at a borderline level for Aβ (both incidence and extent). Thus, our observations detected in a minimal cortical biopsy are in line with previously published data from several PM studies, where the whole brain has been investigated [15, 30].

Most studies assessing ADNC and neuronal population are performed on PM brain tissue, animal models, or cell cultures. We have unique material to study ADNC and neuronal cell counts in surgical brain biopsies from patients with iNPH. Due to the relatively large number of cases that have been sampled during 10 years, we were able to select a relatively homogenous cohort regarding the age. The absolute benefit while assessing surgical samples in contrast to PM tissue is that alterations to be looked for in the tissue are not affected by factors caused by agonal state or PM events. Furthermore, as previously reported, prospective analysis of tissue alteration, in relation to the clinical progression can be carried out [22, 25].

ADNC pathology is associated with cognitive decline and neuronal damage as well as neuronal death, which are observed in AD [10, 16, 28, 38]. These alterations are not expected to be observed in subjects with iNPH. The observation of ADNC in a cortex biopsy is however in line with the observation that a substantial number of subjects with iNPH develop AD over time [20]. Twenty years of experience while assessing tiny brain biopsies obtained during VPS operation of patients with iNPH and clinical prospective studies of these patients have increased our understanding of both iNPH and as well of AD related neurodegeneration. Based on the above, iNPH seems to be an intriguing and reliable model of AD. The histopathological assessment of the brain tissue of iNPH subjects was carried out to assign a patho-anatomical diagnosis, i.e., with or without ADNC. It is thus surprising that this assessment is seldom performed in this setting.

In this study, we assessed the extent of protein expression in the tissue, applying a computerized morphometrical analysis. We chose to measure the SAF for all the proteins, independent of the compartment of expression to facilitate comparison. For the final SAF, pixels of adequate staining intensity were included. Thus, we feel that the methodology for morphometrical analysis is reliable.

The assessment of a surgical biopsy is certainly informative. However, a tiny biopsy represents only a fraction of a brain and does not represent all the neuroanatomical regions with various vulnerabilities displaying different pathologies. Thus, one needs to acknowledge the influence of sampling deficit on all the above.

In conclusion, here we assessed ADNC and neuronal markers in surgical brain biopsies from patients with iNPH. We identified remarkable neuronal preservation but also a substantial depletion of matrix/neuropil within our cohort. These findings are intriguing as they suggest that a loss of matrix/neuropil might be a hallmark lesion in iNPH. This is in line with the cardinal feature of iNPH, enlarged ventricles. Further, the loss of matrix parallel with preservation of neurons, might be an early sign of neurodegeneration. A substantial number of our patients displayed either Aβ or HPτ or both pathologies, even if the extent of HPτ, was low. These observations are indicative of a progressive neurodegenerative process, result in line with the finding that a substantial number of iNPH patients develop dementia. Additionally, females displayed more advanced ADNC and especially HPτ density in their biopsies, in line with the interpretation that the female gender is a risk factor for ADNC and AD. Surprisingly, we noted a positive correlation between neuronal count and Aβ, but only in females. These findings indicate that even subjects with Aβ in cortex might benefit of neuroprotective therapy. Two decades of experience while assessing brain biopsies from subjects with iNPH, certainly suggest that studying this patient category is a reliable model of AD. Our findings further suggest that a histological assessment of a brain biopsy obtained during a VPS procedure should always be carried out as a diagnostic procedure as it reveals information that cannot be obtained otherwise; hence, the obtained information can lead to a more tailored treatment strategy.
